# Supplementary material for: The discovery of novel noncoding RNAs in 50 bacterial genomes
Source: Nucleic Acids Res. 2024 Apr 22;52(9):5152–65. doi: 10.1093/nar/gkae248 (PMC11109978; doi:10.1093/nar/gkae248)
Supplement: gkae248_Supplemental_Files [file gkae248_supplemental_files.zip › Narunsky 50 Genomes RNA Motifs Files.zip!/Publication/79-F_cylindroides/Figures/LRC-79-6-NC_021019_1_IGR_122_1658022_1658234.sto.pdf]

LRC-79-6-NC_021019_1_IGR_122_1658022_1658234.sto.r2r

5′-UAUUUUUCCCUCCGUUUU●UGUU○UUUACUAUACUAUGAGUAUAAACGAAAUGUUAUGACAUUGCAAGCGCUUUUCACACAAYAUUCACAUAUUUUGGUUAU●UUUUUUAAGUUGUUUARCGCUU●CAUCACAUAUUCUUCGCUUAAG○○○
